# HIV-1 Tat-shortened neurite outgrowth through regulation of microRNA-132 and its target gene expression

**DOI:** 10.1186/s12974-016-0716-2

**Published:** 2016-09-15

**Authors:** Pejman Rahimian, Johnny J. He

**Affiliations:** Department of Cell Biology and Immunology, Graduate School of Biomedical Sciences, University of North Texas Health Science Center, 3500 Camp Bowie Blvd, Fort Worth, TX 76107 USA

**Keywords:** HIV-1 Tat, miR-132, MeCP2, BDNF, p250GAP, Neurite outgrowth, Neurotoxicity

## Abstract

**Background:**

Synaptodendritic damage is a pathological hallmark of HIV-associated neurocognitive disorders, and HIV-1 Tat protein is known to cause such injury in the central nervous system. In this study, we aimed to determine the molecular mechanisms of Tat-induced neurite shortening, specifically the roles of miR-132, an important regulator of neurite morphogenesis in this process.

**Methods:**

The relationship between Tat expression and miR-132 expression was first determined using reverse transcription quantitative PCR (qRT-PCR) in Tat-transfected astrocytes and neurons, astrocytes from Tat-transgenic mice, and HIV-infected astrocytes. qRT-PCR and Western blotting were performed to determine Tat effects on expression of miR-132 target genes methyl CpG-binding protein 2, Rho GTPase activator p250GAP, and brain-derived neurotrophic factor. Exosomes were isolated from Tat-expressing astrocytes, and exosomal microRNA (miRNA) uptake into neurons was studied using miRNA labeling and flow cytometry. The lactate dehydrogenase release was used to determine the cytotoxicity, while immunostaining was used to determine neurite lengths and synapse formation. Tat basic domain deletion mutant and miR-132 mimic and inhibitor were used to determine the specificity of the relationship between Tat and miR-132 and its effects on astrocytes and neurons and the underlying mechanisms of Tat-induced miR-132 expression.

**Results:**

Tat significantly induced miR-132 expression, ensuing down-regulation of miR-132 target genes in astrocytes and neurons. miR-132 induction was associated with phosphorylation of cAMP response element-binding protein and required the basic domain of Tat. miRNA-132 induction had no effects on astrocyte activation or survival but was involved in the direct neurotoxicity of Tat. miR-132 was present in astrocyte-derived exosomes and was taken up by neurons, causing neurite shortening.

**Conclusions:**

Tat-induced miR-132 expression contributes to both direct and astrocyte-mediated Tat neurotoxicity and supports the important roles of miR-132 in controlling neurite outgrowth.

## Background

Human immunodeficiency virus type 1 (HIV-1) enters the central nervous system (CNS) during the acute phase of the infection and results in the production of viral proteins and activation of microglia/macrophages and eventually injury to neurons [1, 2]. Before the introduction of combination antiretroviral therapy (cART), HIV-1 infection of the CNS resulted in HIV-1-associated dementia (HAD) in up to 15 % of the infected individuals [3]. The success of cART in the suppression of active HIV replication dramatically reduced the severity of neurocognitive impairments as evident by the decrease in HAD cases to 2 % [4]. Meanwhile, a less severe form of HIV-associated neurocognitive disorders (HAND), termed minor cognitive and motor disorder (MCMD), is being detected in more than 50 % of HIV-infected individuals [5]. Synaptodendritic injury is a positive correlate of MCMD and has become a reliable indicator of cognitive impairment of HIV-infected individuals as opposed to neuronal loss [6–9]. In addition, synaptic loss has been described in the context of neuroinflammation associated with HIV-1 invasion of the CNS [10, 11]. However, the exact underlying molecular mechanisms of HIV-associated synaptodendritic injury and synaptic loss remain largely unknown.

MicroRNA (miRNA) are master translation regulators which generally initiate degradation of messenger RNA (mRNA) and translational repression [12]; they are often dysregulated in diseases [13–15]. Viral infections in particular have been shown to disrupt cellular miRNA profiles and thus modulate viral replication or the transcriptional machinery of the host [16]. In HIV-1 infection, down-regulation of host miRNA has been linked to anti-viral immunity and replication [17, 18]. miRNA are extensively expressed in the CNS and have broadly been studied in differentiation, dendrite growth, and synaptic function of neurons [19–21]. microRNA profiling of the brain tissues of cognitively impaired HIV patients have revealed differential expression signatures of miRNA in the regulation of dendrite formation and branching [22–25]. Among those is miR-132. miR-132 is one of the best defined miRNA in the CNS, and its roles in dendrite morphology and synapse function have been characterized [26, 27], through two extensively studied miR-132 target genes in dendrites: Rho GTPase activator p250GAP [28] and methyl CpG-binding protein 2 (MecP2) [29, 30]. Brain-derived neurotrophic factor (BDNF) is directly and positively regulated at the transcription level by MecP2 and thus can be considered an indirect target of miR-132 [31, 32]. Notably, dysregulation of miR-132 has been reported in a number of psychiatric, neurodevelopmental, and neurodegenerative diseases [33–35]. Outside the CNS, miR-132 has been shown to be highly up-regulated in HIV-infected CD4^+^ T cells [36]. These findings raise the possibility that miR-132 plays important roles in HIV infection and pathogenesis in the CNS.

Tat is indispensable for HIV-1 transcription and replication (see review [37]). Tat is also important for HAND, as it is a potent neurotoxin and causes direct acute damage to neurons in both in vitro and in vivo [38–41]*.* Despite the restricted nature, HIV-1 infection of astrocytes leads to abundant expression of HIV-1 early gene products Tat, Nef, and Rev proteins [42–45]. Tat possesses a unique property, i.e., release from one cell and uptake by the other cell [46, 47]. Consistent with these findings, Tat is present in the CNS of HIV-infected individuals [48, 49] and expressed in and secreted by HIV-1 latently infected astrocytes [50–52]. In addition, Tat adversely affects neuron survival at more physiologically relevant concentrations through cytokine expression, excitatory properties, intracellular signaling, autophagy, and lysoendosomal function in the neurons [53–56]. Importantly, astrocytes have been shown to potentiate Tat neurotoxicity [40, 50, 57, 58]. HIV-1 Tat protein alone is capable of reducing synaptic protein levels and dendritic arbor [59–64]. Of particular note is that Tat expression in astrocytes alone is capable of inducing pathological changes such as loss of axons and dendrites and neurobehavioral changes such as impaired motor and cognitive functions in the CNS reminiscent of HAND [57, 65, 66]. HIV-1 Tat itself has been shown to inhibit anti-viral miRNA expression in peripheral blood mononuclear cells [67]. In the context of HIV-1 infection of the CNS, Tat has been reported to alter miRNA expression in neurons and affect neuronal function [68, 69]. In this study, we used a combined molecular, cellular, and genetic approach and determined the direct relationship between Tat and miR-132 expression and its possible contribution to Tat-induced changes in neurite outgrowth and synapse formation.

## Methods

### Cells and cell cultures

Human kidney epithelial cells 293T, human astrocytoma cells U373.MG and U138.MG, and human neuroblastoma cells SH-SY5Y were purchased from American Type Culture Collection (ATCC, Manassas, VA) and maintained in Dulbecco’s modified Eagle’s medium (DMEM), supplemented with 10 % fetal bovine serum (FBS), 50 U/ml penicillin, and 50 μg/ml streptomycin in a 37 °C, 5 % CO2 incubator. SH-SY5Y were differentiated by sequential treatment with retinoic acid and BDNF as described previously [70] and maintained in 50:50 F-12 HAM/DMEM and 10 % FBS without antibiotics. Primary human astrocytes were prepared as described previously [49]. Primary astrocytes were cultured in an F12-K medium (Cellgro, Manassas, VA) containing 10 % FBS, 50 U/ml penicillin, and 50 μg/ml streptomycin in a 37 °C, 5 % CO2 incubator and passaged every 3–4 days. Primary mouse astrocytes were isolated from 18.5-day-old embryonic brain tissue of the doxycycline-inducible astrocyte-specific Tat-transgenic mice (iTat) or C57B/L6 wild-type mice (WT) as described previously [40]. Primary mouse cortex neurons were isolated from day-17 embryonic C57BL/6 mice, purchased from GIBCO (Langley, OK), and maintained in a Neurobasal medium (GIBCO) supplemented with 1 % B27, 50 U/ml penicillin, and 50 μg/ml streptomycin in a 37 °C, 5 % CO2 incubator.

### Plasmids, miRNA, and transfections

cDNA3 plasmid was purchased from Clontech (Mountain View, CA). Tat.Myc and Tat.His plasmids were previously described [71]. Mutant Tat plasmid with deletion of the basic domain region, aa 49-57 (ΔBD), was constructed using the QuikChange II XL Site-Directed Mutagenesis Kit (Stratagene, La Jolla, CA) with Tat.His as the template and primers 5′-GGC ATC TCC TAT GGC CCT CCT CAA GGA TCC-3′ and 5′-GGA TCC TTG AGG AGG GCC ATA GGA GAT GCC-3′. All recombinant plasmids were verified by sequencing. Synthetic miR-132 mimic (Mission microRNA hsa-miR-132, Cat. HMI0190, mature sequence ACCGUGGCUUUCGAUUGUUACU) and miR-132 inhibitor (Mission microRNA, hsa-miR-132, Cat. HSTUD0191, mature sequence UAACAGUCUACAGCCAUGGUCG) duplexes along with negative control miRNA (Mission microRNA Negative Control 1, Cat. HMC003, mature sequence CGGUACGAUCGCGGCGGGAUAUC) were purchased from Sigma (Sigma-Aldrich, St. Louis, Mo). miR-132 labeling was performed using a Mirus Label IT miRNA Cy^3^ Kit (Madison, WI) according to the manufacturer’s protocol. 239T and U373.MG were transfected using the standard calcium phosphate precipitation method. SH-SY5Y and primary mouse astrocytes were transfected using Lipofectamine 2000 (Invitrogen, Carlsbad, CA). For miRNA transfection, cells were plated and cultured in a 24-well plate and transfected with miR-132 mimic (5 nM/well) and miR-132 inhibitor (50 nM/well) using Lipofectamine 2000 (Invitrogen).

### Reverse transcription quantitative polymerase chain reaction (qRT-PCR)

RNA was isolated from cells or exosomal pellets using a mirVana miRNA Isolation Kit (Ambion, Austin, TX) according to the manufacturer’s protocol. A total of 1 μg RNA was used for reverse transcription using a TaqMan MicroRNA Reverse Transcription Kit (Applied Biosystems, Foster City, CA, USA) followed by qPCR using AmpliTaq Gold PCR Master Mix (Applied Biosystems, Foster City, CA, USA). miR-132 levels were normalized to snRNA U6 (cells), or let-7b (exosomes) [72], calculated as fold change based on the 2^-ddCT^ method, and expressed in log_10_ values. Real-time PCR primers were as follows: for BDNF: 5′-CCT GGT GGA ACT TCT TTG CGG-3′ and 5′-GAA AGC GAG CCC CAG TTT GG-3′; for MecP2: 5′-GGA GCC TGA CCC TTC TGA TG-3′ and 5′-GGA TGT TAG GGC TCA GGG AAG-3′; for CCL2: 5′-CTT CTT TGG GAC ACT TGC TGC-3′ and 5′-CTC AGC CAG ATG CAA TCA ATG-3′; for IL-6: 5′-ACA AAC AAA TTC GGT AGA TCC TCG-3′ and 5′-AGC CAT CTT TGG AAG GTT CAG G-3′; and for TNF-α: 5′-TCT TCT CGA ACC CCG AGT GA-3′ and 5′-CCT CTG ATG GCA CCA CCA G-3′. All real-time PCR assays were run on a Bio-Rad CFX system (Bio-Rad, Hercules, CA). All those mRNA were normalized to β-actin unless stated otherwise.

### Western blotting

Cells were washed twice with ice-cold phosphate-buffered saline (PBS) and lysed in RIPA buffer (50 mM Tris.HCl, pH 7.4, 150 mM NaCl, 1 % Triton X-100, 1 % sodium deoxycholate, 0.1 % sodium dodecyl sulfate (SDS), 2 mM PMSF, and 1× protease inhibitor cocktail (Roche, Indianapolis, IN)) on ice for 20 min for whole cell lysate preparation. Protein concentration was determined using a Bio-Rad DC protein assay kit (Bio-Rad, Hercules, CA), and the optical density at 650 nm was taken using a Bio-Rad iMark microplate reader (Bio-Rad). Cell lysates were electrophoretically separated by 8–12 % sodium dodecyl sulfate-polyacrylamide (SDS-PAGE) gels and Western blot analysis with the following primary antibodies: α-CREB, α-PCREB, α-BDNF (for the mature form of BDNF), α-p250GAP, α-MecP2, α-synaptophysin, α-PSD-95, α-C-MYC, α-CBP (Santa Cruz Biotechnology, Dallas, TX), α-β-actin (Sigma, St. Louis, MO), α-MAP-2 (EMD Millipore, Billerica, MA), α-His (Applied Biological Materials ABM, Richmond, BC), and α-p24 (NIH AIDS Reagents Program, donated by Dr. Bruce Chesebro of NIAID) [73]. Sheep anti-mouse IgG-HRP and donkey anti-rabbit IgG-HRP from GE Healthcare (Marlborough, MA) were used as secondary antibodies, followed by ECL detection and imaging using a Bio-Rad ChemiDoc imaging system (Bio-Rad).

### Immunofluorescence staining

Cells were plated and cultured in a 24-well plate containing poly-lysine-coated coverslips (Sigma). Following transfections and culturing at 37 °C for 48 h, the cells were washed with ice-cold PBS at room temperature for 10 min, fixed in 4 % paraformaldehyde for 30 min, permeabilized in 0.5 % Triton X-100 in PBS for 30 min, blocked in PBS-BB (5 % BSA, 1 % non-fat milk, and 0.3 % Triton X-100 in PBS) for 1 h, stained with a primary antibody (rabbit monoclonal α-MAP-2 (1:1000 in PBS-BB), goat α-SYP (1:250), or mouse α-PSD-95 (1:250)), then stained with a secondary antibody (goat anti-mouse Alexa Fluor 555, goat anti-rabbit Alexa Fluor 488, or sheep anti-goat Alexa Fluor 350, all in 1:2000 in PBS-BB, Invitrogen). Nuclei were counterstained with 0.1 μg/ml 4′, 6-diamidino-2-phenylindole (DAPI). Extensive washes with PBS were performed between each step. Omission of the primary antibody in parallel staining was included as a control to ensure no non-specific staining. Images were taken using a Zeiss Axiovert 200 microscope.

### Lactate dehydrogenase (LDH) release assay

LDH release assay was performed using a Takara LDH Cytotoxicity Detection Kit (Shiga, Japan). Briefly, cells were plated in a 96-well plate and transfected as indicated. Seventy-two hours post transfection, the plate was centrifuged and 150-μl culture supernatant from each well was transferred to a new 96-well plate. Substrate reagent (100 μl) was then added to each well and incubated at 37 °C for 30 min. The plate was immediately placed in a reader and the absorbance at 490 nm was taken. The culture supernatant from cells treated with Triton X-100 was used as a high positive control and media from untreated cells as a low negative control. The media without any cell growth were tested as background. The high positive control was confirmed to have 100 % cell lysis microscopically. Relative LDH release was calculated based on the absorbance of each sample at 490 nm compared with the low and high controls and subtraction of background.

### Exosome isolation

Exosomes were prepared as we described previously [64, 74]. Briefly, the culture supernatants were collected 72 h following transfections and cleared of cell debris by centrifugation at 3000*g* for 10 min followed by filtration through a 0.22-μm filter. Crude exosome pellets were obtained by ultracentrifugation of cleared supernatants at 100,000*g* for 70 min. The exosome pellets were re-suspended in 500 μl PBS and were either frozen at −20 °C or used immediately for uptake studies, RNA isolation, or Western blotting. Exosome-free media were obtained by ultracentrifugation of the complete media (serum with antibiotic) at 100,000*g* for 16 h and used in all studies involving exosomes.

### Virus production and infection

293T cells were transfected with VSV-G and HIV.GFP plasmids using the standard calcium phosphate precipitation method. Culture supernatants were collected 72 h post transfection, cleared of cell debris by low-speed centrifugation and ultracentrifuged through a 6–18 % OptiPrep™ density gradient to purify the HIV virus. The virus titer was determined by the reverse transcriptase assay. For infection of primary human astrocytes and U373.MG (1 × 10^6^), VSV-G-pseudotyped HIV equivalent to 30,000 cpm were added to cells in 6-well plates containing a 2-ml fresh medium and centrifuged at 600*g* in room temperature for 2 h. Cells were then washed with PBS to remove the input virus and were transferred to T25 flasks containing 2 × 10^6^ astrocytes in 18-ml exosome-depleted F-12-K or DMEM. The percentage of infected cells was determined for GFP expression by flow cytometry.

### Data analysis

All experiment data were analyzed by one-way ANOVA unless stated otherwise. A *p* < 0.05 was considered to be statistically significant and marked as “*”, a *p* < 0.01 was considered to be statistically highly significant and marked as “**”, and a *p* < 0.001 was considered to be statistically highly significant and marked as “***.”

## Results

### Tat expression and HIV infection-induced miR-132 expression in astrocytes and neurons

**Fig. 1:**
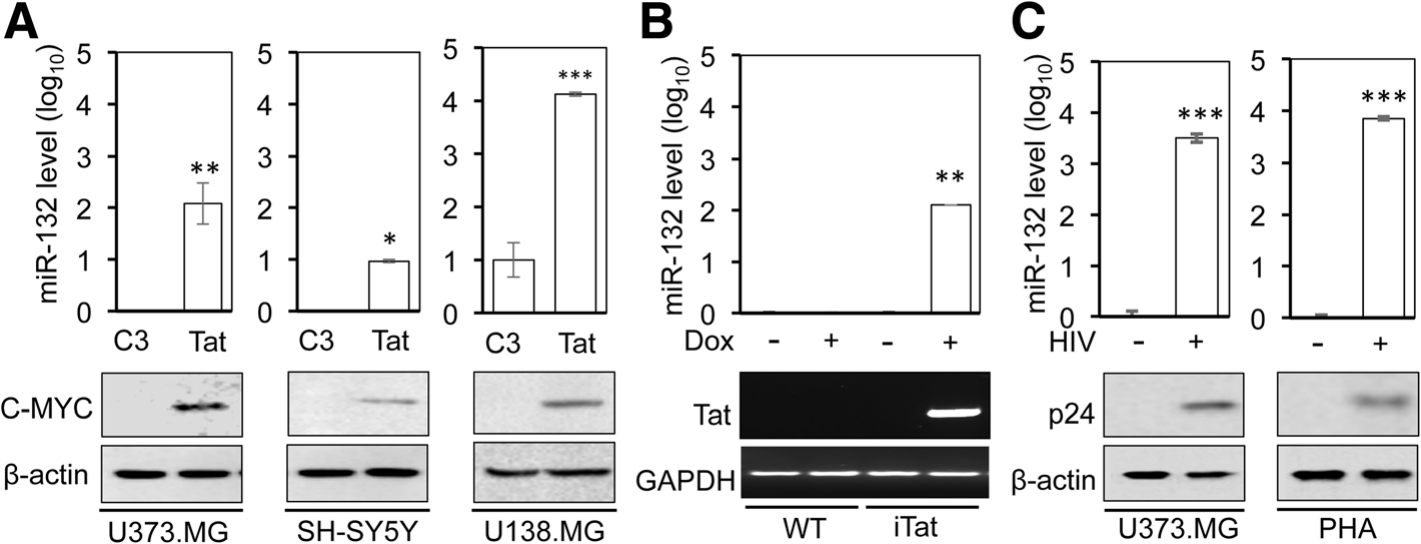
miR-132 induction following Tat expression and HIV-1 infection. **a** U373.MG, SH-SY5Y, and U138.MG were transfected with Tat.Myc plasmid (Tat) or the cloning vehicle pcDNA3 (C3) and cultured for 48 h and harvested for RNA extraction, followed by qRT-PCR for miR-132 level (*upper panels*), or cell lysates, followed by Western blotting for Tat expression using an anti-C-MYC antibody (*lower panels*). β-actin was included as a loading control for Western blotting. **b** Primary astrocytes were isolated from wild-type (WT) mice and doxycycline (Dox)-inducible and astrocyte-specific HIV Tat-transgenic mice (iTat), cultured in the presence of 5 mg/ml Dox (+) or in the absence of Dox (−) for 48 h and harvested for RNA isolation, followed by qRT-PCR for miR-132 level (*upper panel*) or semi-quantitative RT-PCR for Tat (*lower panel*). GAPDH was included as a loading control for Tat RT-PCR. **c** U373.MG and primary human astrocytes (PHA) were infected with VSV-G-pseudotyped HIV (+), cultured for 3 days and harvested for RNA extraction, followed by qRT-PCR for miR-132 level or cell lysates, followed by Western blotting for p24 expression using an anti-p24 antibody. Pseudotyped HIV containing no envelope (−) was included as an infection control (mock). snRNA U6 level was also determined by qRT-PCR. miR-132 level was normalized to snRNA U6 level and expressed as fold changes compared to the control. The data were mean ± SD of triplicates and/or representative of three independent experiments. The data were analyzed using one-tailed Student’s *t* test, and the comparisons were made between the C3 control and Tat

To determine the effects of Tat on miR-132 expression, we first expressed Tat in human astrocytoma cells U373.MG and U138.MG and human neuroblastoma SH-SY5Y and determined miR-132 expression in those cells. Tat expression significantly induced miR-132 level in U373.MG and SH-SY5Y (Fig. 1a). To ascertain Tat-induced miR-132 expression, we took advantage of the doxycycline (Dox)-inducible astrocyte-specific Tat-transgenic mice (iTat) [40, 57], in which Tat expression level and resulting neuropathologies following doxycycline treatment are comparable to those in the brain of HIV-infected subjects. Primary mouse astrocytes from wild-type (WT) and iTat mice were isolated and cultured to induce Tat expression by Dox. miR-132 was significantly induced in Dox-treated astrocytes derived from iTat mice but not in other astrocytes (Fig. 1b). Next, we determined whether miR-132 was induced in astrocytes in the context of HIV infection. U373.MG were infected with VSV-G-pseudotyped HIV. Compared to the mock infection control (HIV containing no envelope), miR-132 was significantly induced in cells infected with VSV-G-pseudotyped HIV (Fig. 1c). Similar results were obtained in primary human astrocytes (PHA) infected with VSV-G-pseudotyped HIV-1. Tat expression was confirmed by Western blotting (Fig. 1a) or semi-quantitative RT-PCR (Fig. 1b), while HIV infection was confirmed by Western blotting for p24 (Fig. 1c). These results show that miR-132 was significantly induced in Tat-expressing astrocytes and neurons and HIV-infected astrocytes.

### Tat expression down-modulated expression of miR-132 target genes

**Fig. 2:**
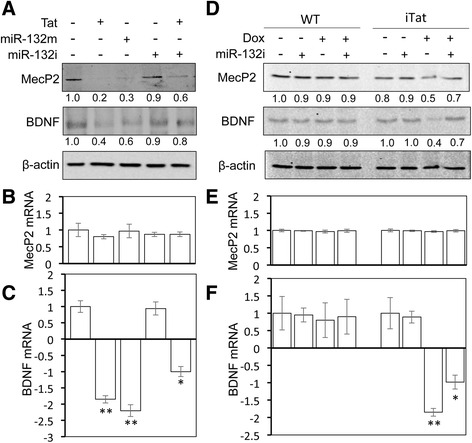
Effects of Tat expression on miR-132 targets in astrocytes. **a–c** Primary human astrocytes were transfected with Tat.Myc, miR-132 mimic (miR-132m), and/or miR-132 inhibitor (miR-132i), cultured for 48 h and harvested for cell lysates, followed by Western blotting (**a**), or RNA isolation, followed by qRT-PCR for MecP2 (**b**) and BDNF (**c**). **d–f** Primary astrocytes were isolated from WT and iTat mice, cultured in the presence (+) or absence (−) of 5 mg/ml Dox for 48 h, transfected with miR-132i, cultured for additional 24 h, and harvested for cell lysates, followed by Western blotting (**d**), or RNA isolation, followed by qRT-PCR for MecP2 (**e**) and BDNF (**f**). A control miRNA and pcDNA3 were included to equalize the input DNA or miRNA. The qPCR data were mean ± SD of triplicates and representative of three independent experiments. Western blots were quantitated using ImageJ software. The relative protein level was calculated using β-actin as the reference, and the first control sample was set at 1.0. The data were representative of three independent experiments

As mentioned above, the mRNA targets of miR-132 within the CNS include MecP2 and p250GAP. BDNF transcription is directly controlled by MecP2, and therefore, BDNF is an indirect target of miR-132. Thus, we determined whether Tat expression altered expression of those miR-132 target genes MecP2, p250GAP, and BDNF. PHA were first used. miR-132 mimic (miR-132m) was included as a control. Both Tat and miR-132 expression led to a lower level of MecP2 and BDNF protein expression in PHA (Fig. 2a). To further determine whether Tat-induced down-modulation of MecP2 and BDNF was mediated through miR-132, we included miR-132 inhibitor (miR-132i) in the experiments. miR-132i expression alone did not significantly affect constitutive MecP2 and BDNF expression but considerably abrogated the down-modulation of MecP2 and BDNF expression by Tat. In addition, MecP2 and BDNF mRNA levels were determined. There were no changes of MecP2 mRNA by Tat, miR-132m, and/or miR-132i (Fig. 2b), while BDNF mRNA exhibited a pattern of changes similar to its protein (Fig. 2c). Then, we determined the relationship between Tat expression and miR-132 target gene expression in primary mouse astrocytes from WT or iTat mice. Compared to WT primary astrocytes, Dox treatment of iTat primary astrocytes led to a lower level of MecP2 and BDNF protein expression (Fig. 2d), little changes in MecP2 mRNA (Fig. 2e), and a low level of BDNF mRNA (Fig. 2f). In parallel, miR-132i expression significantly restored MecP2 and BDNF protein expression and BDNF mRNA expression but had little effects on MeCp2 mRNA expression.

**Fig. 3:**
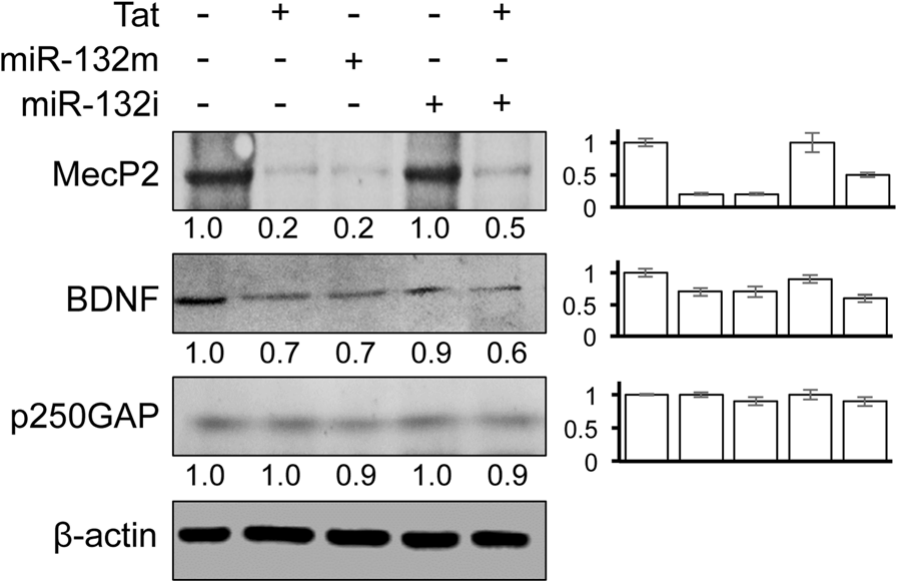
Effects of Tat expression on miR-132 targets in neurons. SH-SY5Y were transfected with Tat.Myc, miR-132 mimic (miR-132m), and/or miR-132 inhibitor (miR-132i), cultured for 48 h and harvested for cell lysates, followed by Western blotting. A control miRNA and pcDNA3 were included to equalize the input DNA or miRNA. Western blots were quantitated using ImageJ software. The relative protein level was calculated using β-actin as the reference, and the first control sample was set at 1.0. The data were representative of three independent experiments

We also assessed the protein levels of miR-132 targets in relation to Tat, miR-132m, and miR-132i expression in SH-SY5Y. In addition to MecP2 and BDNF, the protein level of p250GAP, a neuron-specific target of miR-132, was analyzed. Similar to the findings in astrocytes, there were considerably lower levels of MecP2 and BDNF protein and slightly lower levels of p250GAP protein in Tat- and miR-132m-transfected cells (Fig. 3). miR-132i rescued MecP2 protein expression from Tat-induced down-modulation but had no apparent effects on BDNF and p250GAP expression. Taken together, these results indicate that Tat expression resulted in down-modulation of miR-132 target genes through miR-132 induction.

### Involvement of CREB phosphorylation and Tat basic domain in Tat-induced miR-132 expression

**Fig. 4:**
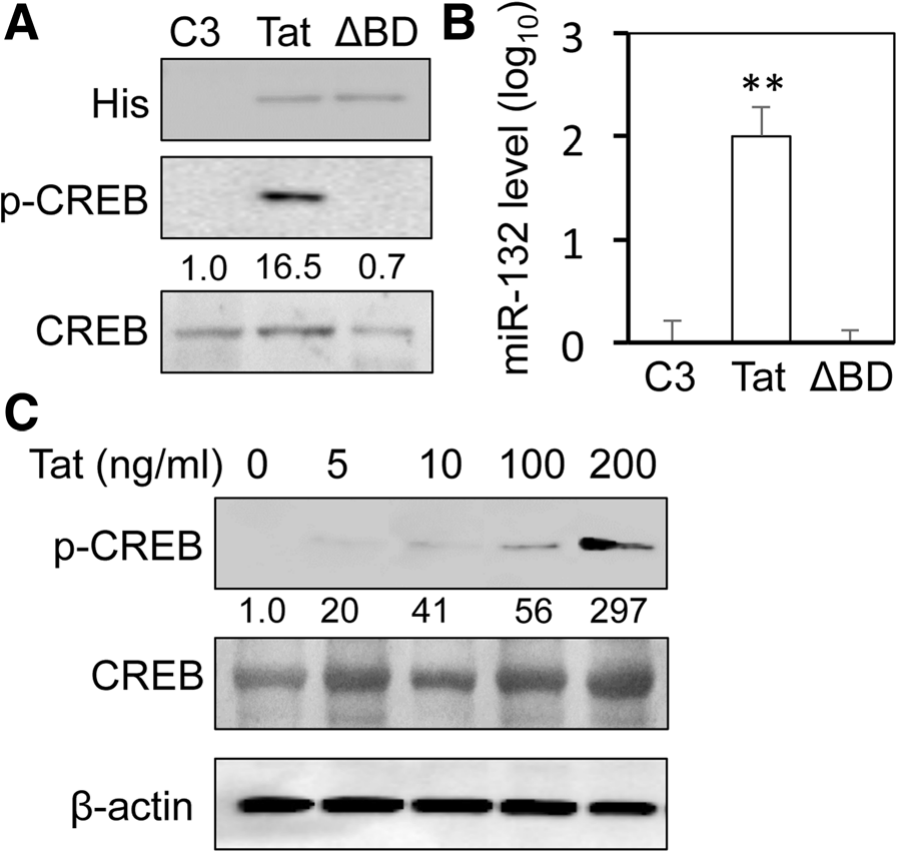
CREB phosphorylation by Tat and its requirement for Tat basic domain. **a**, **b** U373.MG were transfected with Tat.His or basic domain-deleted Tat (ΔBD.His) plasmid, cultured for 48 h and harvested for cell lysates, followed by Western blotting (**a**), or RNA isolation, followed by qRT-PCR for miR-132 level (**b**). C3 was used as the control, and snRNA U6 was used to normalize miR-132 level. A control miRNA was included to equalize the input miRNA. The qPCR data were mean ± SD of triplicates and representative of three independent experiments. **c** Primary human astrocytes were treated with recombinant Tat protein at 0, 5, 10, 100, and 200 ng/ml for 24 h. Cell lysates were then prepared for Western Blotting for CREB and pCREB. β-actin was included as a loading control for Western blotting. Western blots were quantitated using ImageJ software. The relative protein level was calculated using β-actin as the reference, and the first control sample was set at 1.0. The data were representative of three independent experiments

HIV-1 Tat has been shown to regulate transcription of host genes and miRNA through a number of transcriptional factors including CREB [65, 75–82]. miRNA expression is mainly regulated at the level of transcription (see review [83]). The primary sequence, the seed region, and the gene structure of miR-132 are well conserved between humans and mice [27]. A total of five transcriptional binding sites have been characterized within the miR-132 promoter [84–86]: four sites for CREB and one site for repressor element 1 silencing transcription factor/neuron-restrictive factor. Thus, we determined the possibility of the involvement of CREB phosphorylation in Tat-induced miR-132 expression. U373.MG were first used. Tat expression led to phosphorylation of CREB (Fig. 4a) and miR-132 induction (Fig. 4b). Deletion of Tat basic domain (ΔBD) completely abrogated Tat-induced CREB phosphorylation and miR-132 expression. To determine the minimal level of Tat for CREB phosphorylation, PHA were directly exposed to different concentrations of recombinant Tat protein, and CREB phosphorylation was determined using Western blotting. Phosphorylated CREB was detected at 5 ng/ml recombinant Tat protein and increased with increasing concentrations of recombinant Tat protein (Fig. 4c). The minimal level of Tat required for CREB phosphorylation in astrocytes is close to that (up to 16 ng/ml) reported in the CSF of HIV patients [87].

**Fig. 5:**
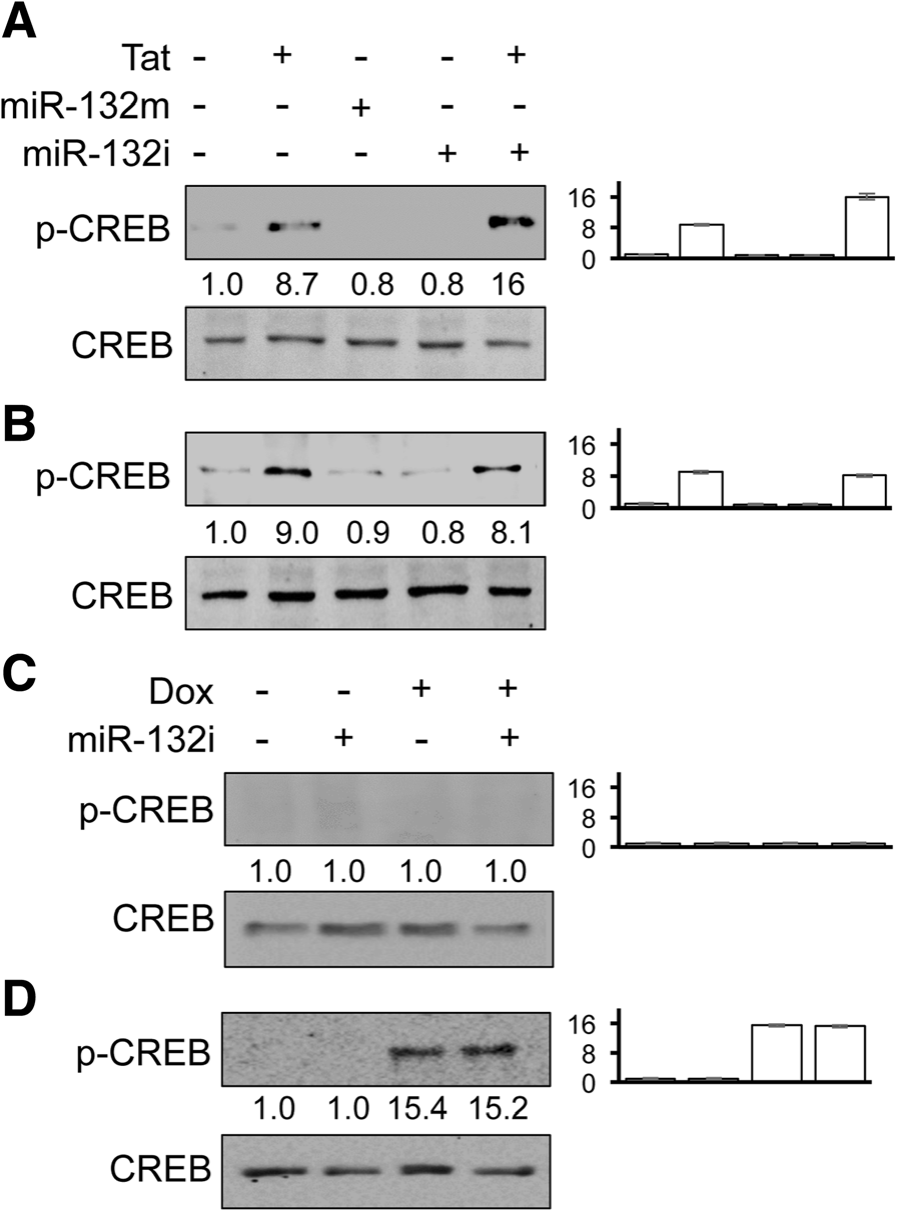
CREB phosphorylation and miR132 expression. **a**, **b** Primary human astrocytes (**a**) and SH-SY5Y (**b**) were transfected with Tat, miR-132m, and/or miR-132i, cultured for 48 h and harvested for cell lysates, followed by Western blotting. A control miRNA and pcDNA3 were included to equalize the input DNA or miRNA. **c**, **d** Primary astrocytes were isolated from WT and iTat mice, cultured in the presence (+) or absence (−) of 5 mg/ml Dox for 48 h, transfected with miR-132i, cultured for additional 24 h, and harvested for cell lysates, followed by Western blotting. A control miRNA was included to equalize the input miRNA. Western blots were quantitated using ImageJ software. The relative protein level was calculated using β-actin as the reference, and the first control sample was set at 1.0. The data were representative of three independent experiments

Then, to determine the role of CREB phosphorylation in Tat-induced miR-132 expression, PHA and SH-SY5Y were transfected with Tat, along with miR-132m or miR-132i. In PHA, Tat expression induced CREB phosphorylation (Fig. 5a). Although miR-132m and miR-132i expression did not lead to significant changes in constitutive CREB phosphorylation, co-expression of Tat and miR-132i resulted in a much higher level of CREB phosphorylation. In SH-SY5Y, Tat expression induced CREB phosphorylation (Fig. 5b) although both miR-132m and miR-132i showed no effects on the constitutive level of CREB phosphorylation. Furthermore, primary astrocytes from WT and iTat mice were also used to assess the relationship between Tat expression and CREB phosphorylation. Compared to the WT astrocytes (Fig. 5c), iTat astrocytes treated with Dox led to CREB phosphorylation (Fig. 5d), which was not affected by miR-132i expression. These results together suggest that CREB phosphorylation and Tat basic domain are likely involved in Tat-induced miR-132 expression.

### miR-132 induction did not contribute to Tat-induced astrocyte activation

**Fig. 6:**
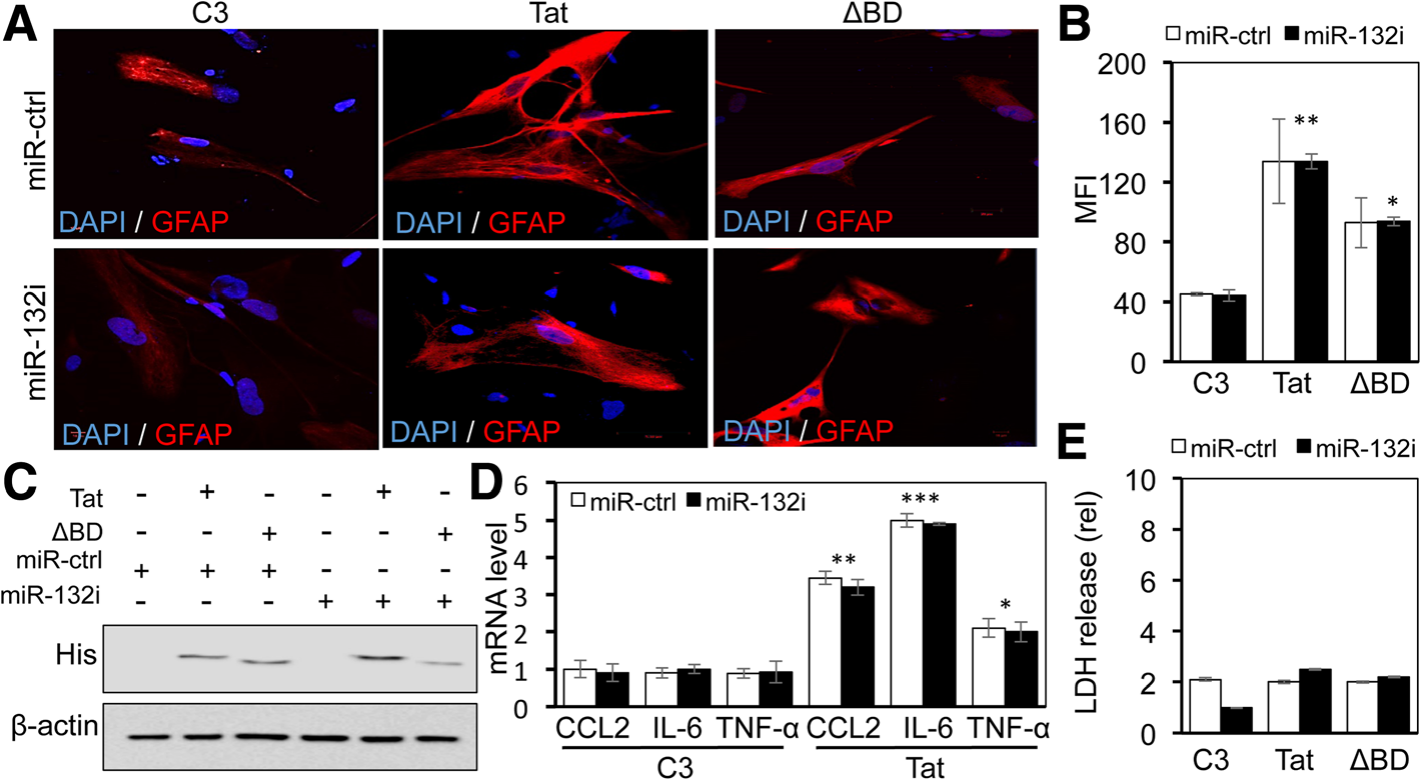
Effects of miR-132 induction on GFAP expression, cytokine/chemokine expression, and astrocyte survival. Primary human astrocytes were transfected with Tat.His (*Tat*), ΔBDTat.His (*ΔBD*), and miR-132i (*miR-132i*), cultured for 48 h and harvested for immunofluorescence staining for GFAP (*red*) and DAPI staining for nucleus (*blue*) (**a**), Western blotting (**c**), or qRT-PCR (**d**). The mean fluorescence intensity (MFI) of GFAP expression was determined using ImageJ software (**b**). Meanwhile, the cell culture supernatants were collected and assayed for the LDH release (**e**). The data (**b**, **d**, **e**) were mean ± SD of triplicates and representative of three independent experiments

Tat expression in astrocytes induces a highly inflammatory profile and leads to the dysregulation of astrocyte functions, termed astrocyte activation, or astrocytosis [65, 88], characterized by increased GFAP expression [88] and expression of pro-inflammatory cytokines such as IL-6 and TNF-α and chemokines such as CCL2 [89–91]. Next, we determined whether miR-132 induction was involved in Tat-induced GFAP, CCL-2, IL-6, and TNF-α expression. As shown previously [57], Tat expression induced GFAP expression in PHA when compared to the C3 control (Fig. 6a, b). ΔBD Tat mutant also induced GFAP expression albeit to a lesser extent, and miR-132i did not show any effect on GFAP expression induced by Tat or ΔBD. Tat and ΔBD expression was confirmed by Western blotting (Fig. 6c). Similarly, Tat induced CCL-2, IL-6, and TNF-α expression when compared to the C3 control (Fig. 6d). But miR-132i had little effects on constitutive and Tat-induced expression of those molecules. In addition, we also determined effects of Tat on the LDH release, an indicator of cell viability. Compared to the C3 control, Tat or ΔBD expression showed little effects on the LDH release (Fig. 6e). Taken together, these results suggest that miR-132 induction is unlikely involved in Tat-induced astrocyte activation and that Tat expression itself does not affect astrocyte survival.

### Tat-induced miR-132 expression contributed to Tat direct neurotoxicity

**Fig. 7:**
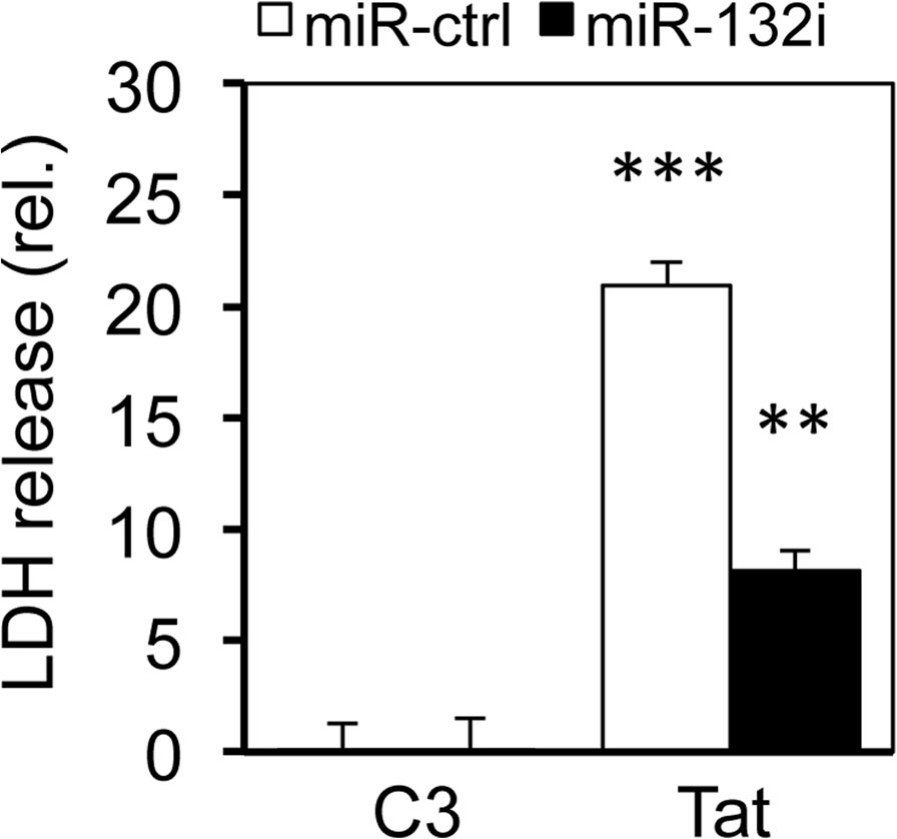
Role of miR-132 induction in Tat neurotoxicity. SH-SY5Y were transfected with Tat.Myc (*Tat*), miR-132i, or both and cultured for 48 h. The cell culture supernatants were collected and assayed for the LDH release. *C3* and a control miRNA (*miR-ctrl*) were used to equalize the input amount of DNA and miRNA. The data were mean ± SD of triplicates and representative of three independent experiments

To determine whether Tat-induced miR-132 contributed to Tat direct neurotoxicity, SH-SY5Y were transfected with Tat and miR-132i and assayed for the LDH release from those cells. Compared to the C3 control, Tat expression increased the LDH release from those cells (Fig. 7). Meanwhile, miR-132i expression significantly diminished Tat-induced LDH release from those cells. These results confirm that Tat has direct neurotoxic activity and suggest that miR-132 induction could contribute to Tat neurotoxicity.

### Tat expression led to increased miR-132 level in astrocyte-derived exosomes

**Fig. 8:**
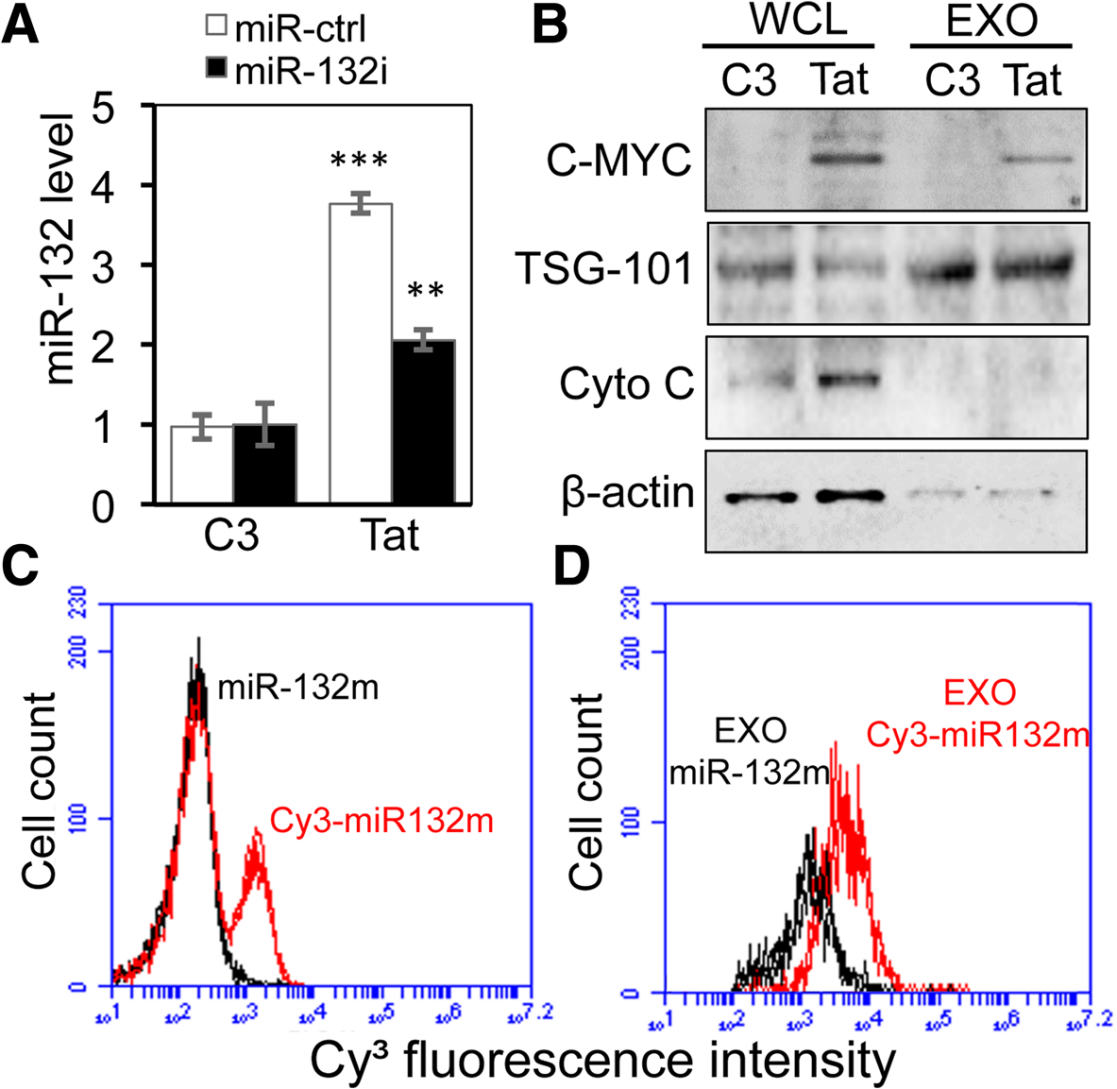
Exosomal miR-132 and its transfer to neurons. **a**, **b** U373.MG were transfected with Tat, miR-132i, or both and cultured for 48 h. The cell culture supernatants were collected and used to isolate exosomes. RNA was isolated from exosomes and analyzed for qRT-PCR for miRNA-132 level (**a**). Exosomal miR-132 was normalized to let-7b. Lysates were prepared from the cells (WCL) and exosomes (EXO) and analyzed by Western blotting (**b**). **c, d** U373.MG were transfected with Cy^3^ dye miR-132m (Cy^3^-miR-132m) or unlabeled miR-132m as a control, cultured for 12 h, and harvested for Cy^3^ signal using flow cytometry (**c**). The cell culture supernatants were collected and used to isolate exosomes. SH-SY5Y were cultured in the presence of the exosomes for 6 h and assayed for Cy^3^ signal using flow cytometry (**d**). The qRT-PCR data were mean ± SD of triplicates and representative of three independent experiments. The Western blots and the flow cytometry histograms were representative of three independent experiments

Virtually all eukaryotic cells including astrocytes produce exosomes, which serve as a major extracellular vehicle to transport miRNA and proteins among cells [13, 72]. Thus, we first determined whether Tat-induced miR-132 expression would lead to increased miR-132 in astrocyte-derived exosomes. Compared to the C3 control, increased miR-132 was detected in exosomes derived from Tat-expressing cells (Fig. 8a). miR-132i expression significantly decreased Tat-induced miR-132 packaging into the exosomes from astrocytes, further supporting the notion that miR-132 is sorted into the exosomes. The exosome purity was confirmed by lack of cytochrome C and presence of TSG-101 in the exosomes using Western blotting (Fig. 8b). Consistent with our previous studies [64], Tat was also detected in the exosomes. To determine whether miR-132 in the form of exosomes could be transferred from astrocytes to neurons, we labeled miR-132m with Cy^3^ and transfected it into U373.MG and further confirmed Cy^3^-labled miR-132m transfection by flow cytometry (Fig. 8c). Furthermore, we obtained exosomes from Cy^3^-labled miR-132m-transfected astrocytes and exposed them to SH-SY5Y and determined whether those cells would take up the labeled miRNA by flow cytometry. Cy^3^-labled miR-132m were detected in those cells (Fig. 8d). These results show that Tat-induced miR-132 is packaged into astrocyte-derived exosomes, which can be taken up by neurons.

### Astrocyte-derived miR-132 shortened neurites

**Fig. 9:**
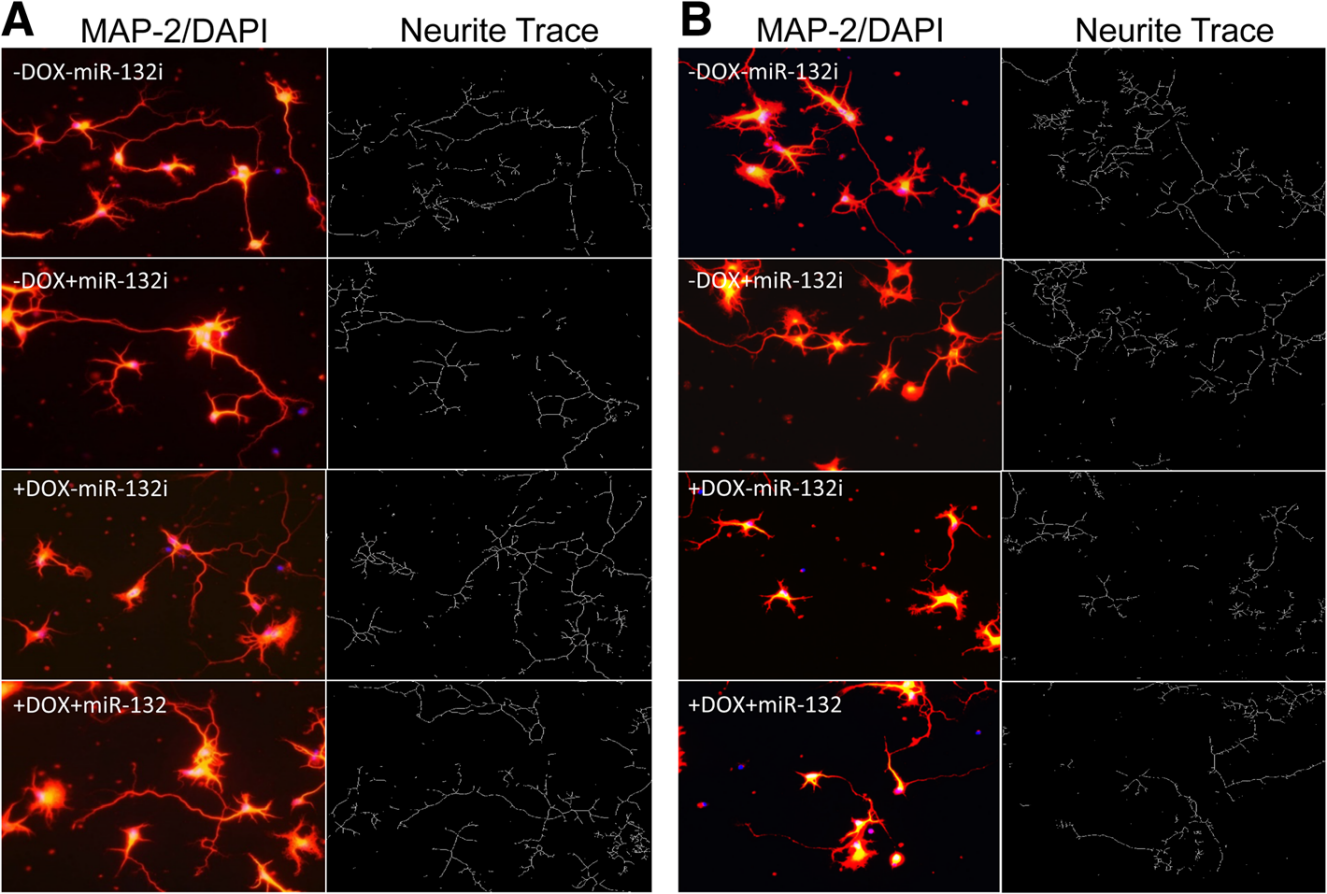
Effects of Tat expression and exosomal miR-132 induction on neurite outgrowth. Primary mouse astrocytes were isolated from WT (**a**) and iTat (**b**), cultured in the presence (+Dox) or absence of (−Dox) of 5 mg/ml for 48 h, transfected with miR-132i (+miR-132i) or a control miRNA (−miR-132i) and continued to culture for 48 h. The cell culture supernatants were collected and used to isolate exosomes. Primary mouse cortical neurons were plated and cultured on poly-lysine-coated coverslips in a 24-well plate at the density of 85,000 cells/well and continued to culture in the presence of the exosomes isolated above for 48 h. Then, the cells were harvested for immunofluorescence staining for MAP-2 and counterstained with DAPI (**a**, **b**). The images were representative of three independent experiments

**Fig. 10:**
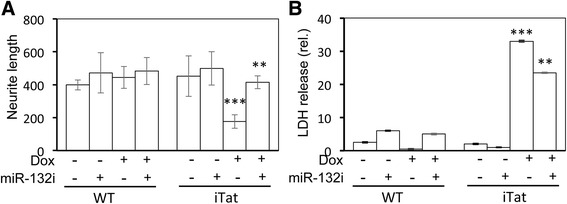
Changes of neurite lengths and neuron survival by Tat and exosomal miR-132. The average length of neurites in Fig. 9a, b above was determined using ImageJ Neurite Tracer and calculated based on the occupied pixel areas and three randomly selected images of each treatment group (**a**). The cell culture supernatants from Fig. 9a, b above were collected and assayed for LDH release (**b**). The data were representative of three independent experiments

**Fig. 11:**
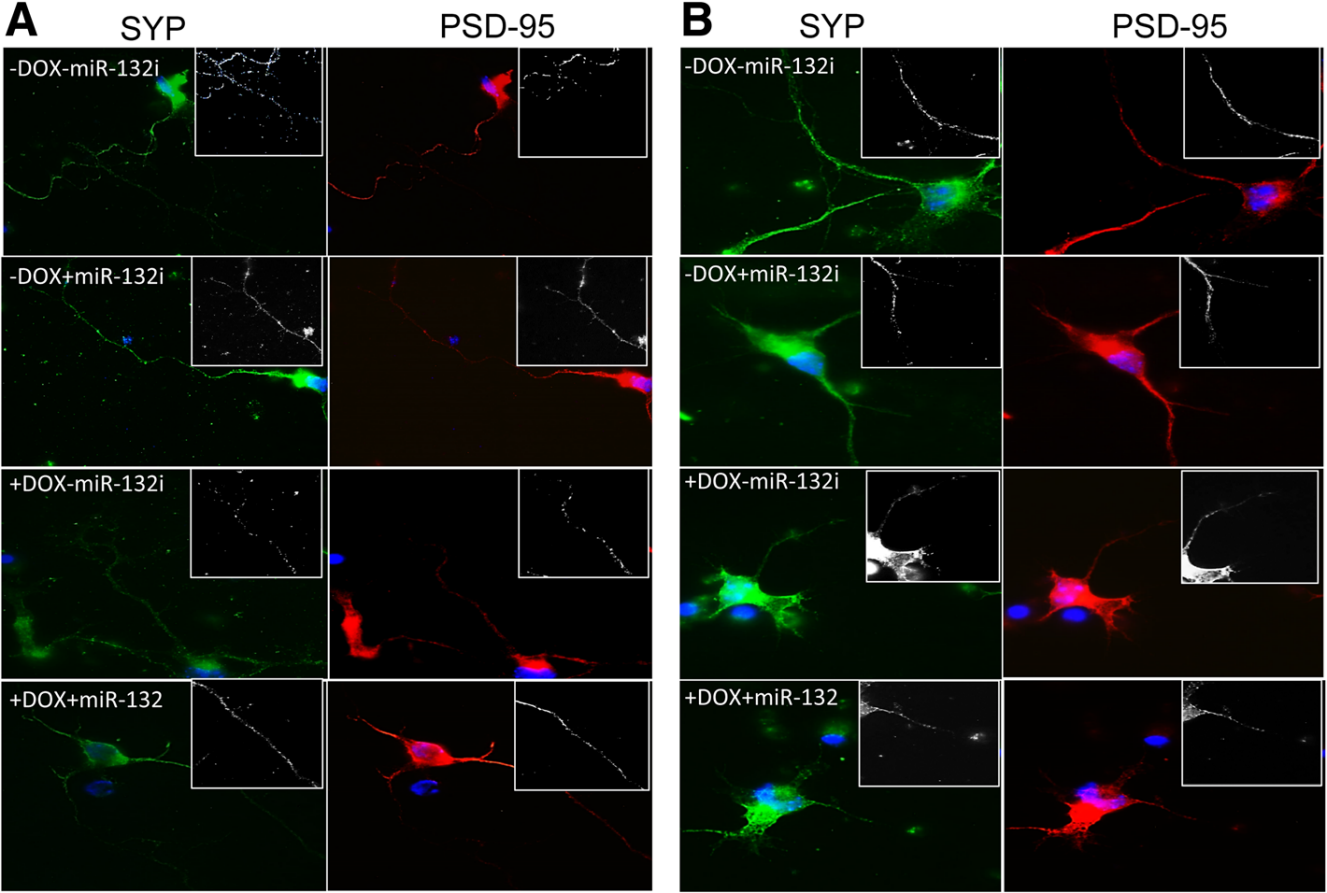
Effects of Tat expression and exosomal miR-132 induction on synaptic formation. Primary mouse astrocytes were isolated from WT (**a**) and iTat (**b**), cultured in the presence (+Dox) or absence of (−Dox) of 5 mg/ml for 48 h, transfected with miR-132i (+miR-132i) or a control miRNA (−miR-132i) and continued to culture for 48 h. The cell culture supernatants were collected and used to isolate exosomes. Primary mouse cortical neurons were plated and cultured on poly-lysine-coated coverslips in a 24-well plate at the density of 85,000 cells/well and continued to culture in the presence of the exosomes isolated above for 48 h. The cells were then harvested for immunofluorescence staining for synaptophysin (SYP) for presynapse formation and for PSD-95 for postsynapse formation and counterstained with DAPI (**a**, **b**). The images were representative of three independent experiments

**Fig. 12:**
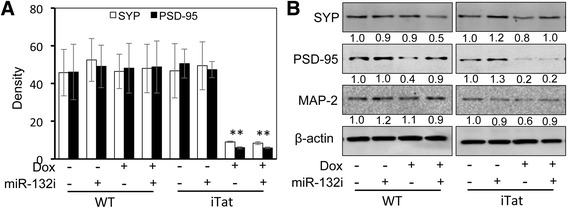
Changes of SYP and PSD-95 expression by Tat and exosomal miR-132. SYP and PSD-95 immunostaining along with skeleton conversion of fluorescent images in Fig. 11a, b above was performed, the density of synaptic protein staining was determined using ImageJ software and based on the occupied pixel areas and three randomly selected images of each treatment group (**a**). The cells from Fig. 11a, b above were harvested for cell lysates, followed by Western blotting (**b**). Western blots were quantitated using ImageJ software. The relative protein level was calculated using β-actin as the reference, and the first control sample was set at 1.0. The data were representative of three independent experiments

We next sought to determine whether transfer of exosome-associated miR-132 from astrocytes to neurons would affect the morphology of neuron dendrites and formation of synapse. To this end, WT and iTat primary astrocytes were prepared, cultured in the presence or absence of Dox, and transfected with miR-132i, and exosomes were prepared from those samples and exposed to primary mouse cortical neurons. The primary cortical neurons were stained for MAP-2, and the neurite length of the primary cortical neurons that were exposed to exosomes from WT astrocytes (Fig. 9a) and exosomes from iTat astrocytes (Fig. 9b) was quantitated using the neurite trace analysis. Compared to the WT controls, exosomes from Dox-treated iTat astrocytes showed significantly shorter neurites (Fig. 10a), which was significantly reversed by miR-132i expression. In parallel, exosomes from Dox-treated iTat astrocytes increased the LDH release from the neurons, which was significantly diminished by miR-132i expression (Fig. 10b). Meanwhile, the exosome-treated primary cortical neurons were also stained for synaptophysin (SYP), a pre-synaptic marker, or postsynaptic density protein (PSD-95), a postsynaptic marker. The density of SYP and PSD-95 of the primary cortical neurons that were exposed to exosomes from WT astrocytes (Fig. 11a) and exosomes from iTat astrocytes (Fig. 11b) was similarly quantitated. Compared to the WT controls, exosomes from Dox-treated iTat astrocytes significantly decreased the density of both SYP and PSD-95 in those cells (Fig. 12a), which was not rescued by miR-132i expression. Similar results were obtained by Western blotting (Fig. 12b). These results showed that Tat-induced miR-132 in astrocytes adversely affected neurite outgrowth and contributed to astrocyte-mediated Tat neurotoxicity.

## Discussion

HIV-1 Tat damages the dendritic arbor, shortens neurites, and reduces synaptic protein levels [59, 60, 63]. Widespread expression and secretion of this protein in the CNS of the HIV-infected population despite HAART [51] is strongly correlated with synaptodendritic damage, the hallmark of pathology in HAND [9, 92]. The reversible nature [93] of Tat’s deleterious effects on dendritic integrity warrants a better understanding of the potential molecular events that connect Tat to the regulation of synaptodendritic plasticity. In this study, we first determined the potential of Tat in the induction of miR-132, a brain-enriched microRNA with substantial roles in the regulation of synaptodendritic plasticity. We found that Tat significantly induces this miRNA in astrocytes and neurons (Fig. 1), leading to the repression of the known targets of this miRNA (Figs. 2 and 3), and causing neurotoxicity (Fig. 7). We showed the CREB-responsive miR-132 expression through the phosphorylation of CREB by Tat (Fig. 5). We ruled out the possibility of miR-132 involvement in Tat activation of astrocytes (Fig. 6). Considering the significance of exosomal glia-neuron communications [94] and the direct link between the miRNA processing pathway and exosomal biogenesis [95], we determined the miR-132 content of Tat-expressing astrocyte exosomes. We found significant levels of miR-132 in exosomes from these astrocytes which were taken up by neurons (Fig. 8) and resulted in neurite shortening (Fig. 9 and 10) but were not directly involved in reduction of synaptic protein levels by Tat (Figs. 11 and 12). These data provide new mechanistic insights of Tat’s injury to dendritic arbor and identify novel pathways that potentially contribute to Tat neurotoxicity.

We showed that HIV-1 Tat protein significantly up-regulated miR-132 in astrocytes and neurons (Fig. 1). Unlike an early report [96], our data did not show a difference in the basal expression levels of this microRNA between the two cell types. Following Tat expression, however, astrocytes showed significantly higher fold changes in miR-132 compared to neurons. This might in part be due to the well-documented activity-dependent nature of miR-132 expression in neurons which happens rapidly and in a transient fashion [97]. On the other hand, Tat transfection of neuronal cells was less efficient compared with astrocytes as supported by our Western blotting results which showed a lower level of Tat expression in neurons. We then demonstrated that miR-132 induction was likely due to the ability of Tat to induce phosphorylation of CREB (Fig. 5) and subsequent binding of phosphorylated CREB to CREB-responsive elements within the transcriptional control locus of miR-132 [84]. CREB phosphorylation also promotes the binding of phosphorylated CREB to CBP, which leads to the transactivation of the miR-132 loci containing CREB-responsive elements [98]. Tat expression leads to phosphorylation of CREB at Ser^133^ through several pathways [78, 99, 100]. Alternatively, Tat directly binds CBP, recruiting the histone acetyltransferase to the CRE [82]. Both CREB phosphorylation and CBP-binding activities of Tat have been shown to be dependent on the basic domain of this protein [101, 102]. Our data showed complete abrogation of CREB phosphorylation and lack of miR-132 induction following transfection with basic domain-deleted Tat (Fig. 4a, b), further supporting Tat effects on CREB phosphorylation and the requirement of Tat basic domain for this effect. Despite a comparable level of Tat-induced CREB phosphorylation in both astrocytes (Fig. 5a) and neurons (Fig. 5b), the basal levels of total CREB and phosphorylated CREB in neurons were higher than those in astrocytes. This difference is likely due to the fact that there are more constitutive CREB activation pathways in neurons than astrocytes such as calcium signaling-associated and B-raf kinase-mediated MAPK-dependent phosphorylation of CREB [103–105].

miR-132 plays a crucial role in the development of neurons by regulating two targets in charge of controlling neurite outgrowth: MecP2, which is a potentiating factor of neurite growth, and p250GAP, which inhibits the outgrowth of neurites [27, 106]. The question is then which direction miR-132 expression takes the neurons in regard to neurite growth while this microRNA represses both an enhancer and an inhibitor of neurite outgrowth. The different temporal expression of p250GAP and MecP2 in development may likely provide the key. p250GAP is involved in neuronal differentiation and is expressed prior to MecP2 [107]. In fact, the studies that discovered p250GAP as a target of miR-132 only found repression of this target in immature neurons [84] and showed that miR-132 overexpression led to the enhancement of neurite outgrowth due to the repression of p250GAP. Studies performed on adult neurons, however, showed the complete opposite; overexpressing miR-132 in adult neurons leads to a significant decrease in dendritic growth [108], which is due to MecP2 repression. MecP2 is expressed starting at the 10th gestational week and is required for the maintenance of adult neurons [109]. Our data are in agreement with these findings indicating the repression of MecP2 in fully differentiated neuronal cells while p250GAP levels are unchanged (Fig. 3). p250GAP expression in adult neurons has been found to be activity dependent and controlled by NMDA receptor signaling events [107].

MecP2 has significant roles in neurogenesis, neuronal differentiation, and proper development of dendritic arbor [110, 111]. This major target of miR-132 in the CNS has been found to be dysregulated in HAND [112]. BDNF, another important regulator of dendritic growth [113, 114] and synaptic function [115, 116] which shows notable reduction in the brain and serum of HIV-infected subjects [117, 118], is directly and positively regulated by MecP2 [119]. In fact, an axis of regulation exists in the brain involving BDNF, miR-132, and MecP2 [29], in which BDNF induces miR-132 through phosphorylation of CREB [120, 121]. As a target of miR-132, MecP2 is ultimately repressed, leading to the repression of BDNF due to the negative regulatory feedback nature of this axis. Improper timing or duration of miR-132 expression has been shown to be neurotoxic, indicating the delicate balance in the regulation of this axis [122]. Rett syndrome, an autism spectrum disorder resulting from genetic mutation in the MecP2 gene [123], shares striking similarities in neuropathology with HAND. MecP2 is essential for the proper outgrowth of dendrites and formation of synapses [111]. The activity-dependent expression of this protein starts prior to synaptogenesis in development and is tightly controlled in most adult neurons [124]. Reduced size and branching of dendrites inflicted by the lack of MecP2 expression in the developing brain result in microcephaly and mental retardation. Interestingly, RNA levels of MecP2 do not differ in fetal and adult brains while its expression is not similar in different neuronal populations [125]. This is indicative of posttranscriptional control of MecP2 by miRNA and is in agreement with our data that failed to find changes in MecP2 mRNA but showed significant reduction in MecP2 protein and consequently BDNF, the transcription of which depends on MecP2 (Figs. 2 and 3).

To discern the direct detrimental effects of Tat on neurons from miR-132 overexpression, we employed synthetic miR-132 inhibitor in our experiments. We confirmed that miR-132 inhibitor transfection efficiently antagonized miR-132 (data not shown). Next, we showed that miR-132 overexpression was at least in part responsible for Tat neurotoxicity (Fig. 7). Antagonizing miR-132 expression also led to reduced exosomal levels of miR-132 (Fig. 8a). In our previous study, we reported exosome-associated Tat release from Tat-expressing and HIV-1 infected cells [64]. With miR-132 inhibitor expression, we were able to conclude that overexpression of miR-132 was responsible for neurite shortening in neurons treated with exosomes from Tat-expressing astrocytes (Fig. 10a), as antagonizing miR-132 improved neurite lengths even in presence of Tat-containing exosomes. Similarly, we also showed that exosomal miR-132 contributes to neurotoxicity (Fig. 9b). Although miR-132 inhibitor expression consistently negated the Tat-induced down-modulation of MecP2 in astrocytes (Fig. 2a, d) and neurons (Fig. 3), its effects on rescuing BDNF levels were not consistent across cell types. Only astrocytes (Fig. 2a, d) showed improved BDNF levels with miR-132i transfection, while neurons (Fig. 3) did not show such an effect. This might be due to the temporal difference that exists in MecP2-induced BDNF expression. Antagonizing miR-132 in presence of Tat rescues MecP2 levels as the direct target of miR-132. This process rapidly improves BDNF mRNA levels (Fig. 2c, f), while this improvement is not reflected in protein levels of BDNF as rapidly.

miR-132 has been shown to increase the width of dendritic spines, which are storage sites for post synaptic density, contain glutamate receptors, and increase the contact points between neurons [108]. These protrusions also establish contact with synaptic terminals containing synaptophysin [126]. Therefore, it has been suggested that miR-132 may affect the strength of excitatory synapses [127]. Our analysis of synaptic protein levels in cortical neurons treated with exosomes derived from Tat-expressing astrocytes (+DOX − miR-132i/+DOX + miR-132i) reduced both SYP and PSD-95. Moreover, reducing miR-132 levels in exosomes did not change the significant loss of these proteins (Figs. 11a, b and 12a). Western blotting data from these neurons also showed consistent reduction in both SYP and PSD-95, and although there was no change in PSD-95 with miR-132i, SYP levels showed improvement with reduced exosomal miR-132 (Fig. 12b). However, lack of consistency between the effects of miR-132 inhibition on the two proteins and reduction in protein levels of neurons treated with WT exosomes prevent us from concluding that miR-132 affected synaptic protein levels or synapse formation. MAP-2 protein levels were consistent with the miR-132-dependent reduction in neurite lengths by Tat and increase in neurite lengths by miR-132 inhibition despite of Tat expression. These results are in agreement with Tat’s direct involvement in the reduction of postsynaptic proteins followed by negative feedback [59, 61] on pre-synaptic proteins.

**Fig. 13:**
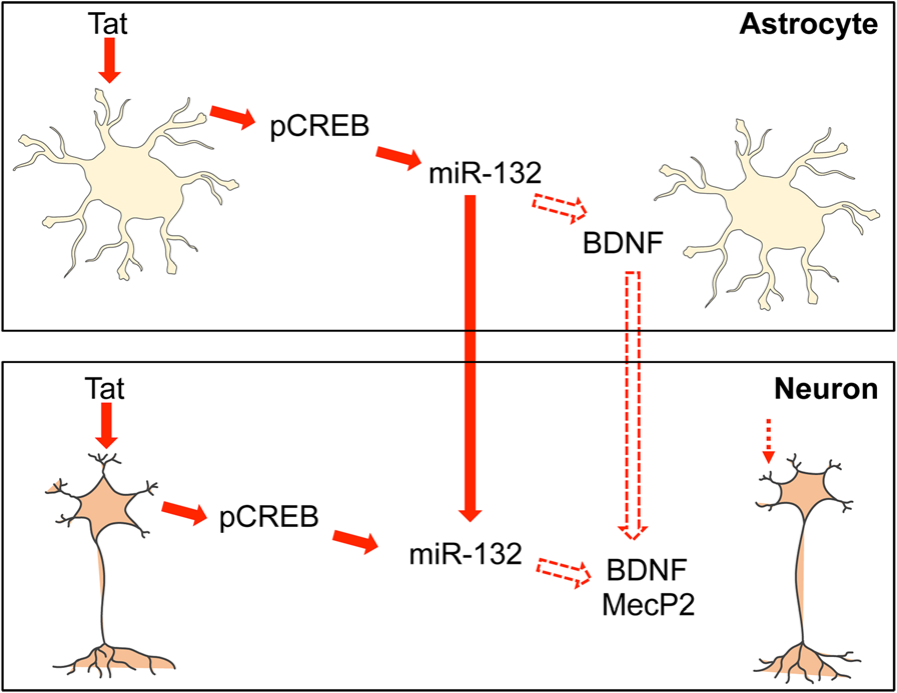
A new model for Tat-impaired neurite outgrowth. Tat impairs neurite outgrowth through its uptake into neurons, CREB phosphorylation (pCREB), miR-132 induction, and MecP2 and BDNF down-regulation. Alternatively, Tat-impaired neurite outgrowth results from HIV-1 infection and Tat expression in astrocytes, pCREB, miR-132 induction, and BDNF down-regulation. miR-132 induction in astrocytes increases exosome-associated miR-132 release from astrocytes, uptake of exosome-associated miR-132 into neurons, and MecP2 and BDNF down-regulation of MecP2 in neurons. In addition, BDNF down-regulation in astrocytes deprives neurons of BDNF, which further impairs neurite outgrowth and neuron survival

In summary, the findings from the current study support a new model of Tat-impaired neurite outgrowth and neuron survival (Fig. 13). Tat-impaired neurite outgrowth could be direct and astrocyte mediated. Tat uptake into neurons induces miR-132 expression through CREB phosphorylation, down-regulates MecP2 and BDNF expression in neurons, and impairs neurite outgrowth and neuron survival; alternatively, Tat expression in astrocytes resulting from HIV-1 infection or Tat uptake into astrocytes induces miR-132 expression through CREB phosphorylation and down-regulates BDNF expression. miR-132 induction in astrocytes leads to increased miR-132 release into exosomes from astrocytes, uptake of exosome-associated miR-132 into neurons, and down-regulation of MecP2 and BDNF expression in neurons and impairs neurite outgrowth and neuron survival. In addition, BDNF down-regulation in astrocytes results in less BDNF available for neurons and adversely affects neurite outgrowth and neuron survival.

## Conclusions

In this study, we report for the first time the induction of miR-132 by Tat and its contribution to Tat-induced neurite shortening and neurotoxicity. The results show that Tat induces miR-132 by activating CREB, the transcriptional control locus of miR-132. We also show that Tat-induced miR-132 leads to the repression of MecP2 and BDNF, known targets of miR-132 in the brain. Correspondingly, the miR-132 level in exosomes from Tat-expressing astrocytes shows significant increase and leads to neurite shortening and neurotoxicity following uptake in neurons.

## Abbreviations

ΔBD: Basic domain-deleted Tat mutant
BDNF: Brain-derived neurotrophic factor
cART: Combination antiretroviral therapy
CNS: Central nervous system
CREB: cAMP response element-binding protein
pCREB: Phosphorylated CREB
Dox: Doxycycline
GFAP: Glial fibrillary acidic protein
HAD: HIV-1-associated dementia
HAND: HIV-associated neurocognitive disorders
HIV-1: Human immunodeficiency virus type 1
iTat: Dox-inducible astrocyte-specific Tat-transgenic mouse
LDH: Lactate dehydrogenase
MAP-2: Microtubule-associated protein 2
MCMD: Minor cognitive and motor disorder
MecP2: Methyl CpG-binding protein
miR (miRNA): microRNA
miR-132: microRNA-132
miR-132i: miR-132 inhibitor
miR-132m: miR-132 mimic
p250GAP: Rho GTPase-activating protein 32
PHA: Primary human astrocytes
PSD-95: Postsynaptic density protein 95
SYP: Synaptophysin
Tat: Transactivator of transcription
WT: Wild-type
